# Growth, Physiology and Nutrient Use Efficiency in *Eugenia dysenterica* DC under Varying Rates of Nitrogen and Phosphorus

**DOI:** 10.3390/plants9060722

**Published:** 2020-06-08

**Authors:** Daniele Nogueira dos Reis, Fabiano Guimarães Silva, Reginaldo da Costa Santana, Thales Caetano de Oliveira, Mariângela Brito Freiberger, Fábia Barbosa da Silva, Elídio Monteiro Júnior, Caroline Müller

**Affiliations:** 1 Plant Tissue Culture Laboratory, Goiano Federal Institute of Science and Technology—Campus Rio Verde, P.O. Box 66, 75901-970 Rio Verde, GO, Brazil; daniele.nog@gmail.com (D.N.d.R.); reginaldocosantana@gmail.com (R.d.C.S.); thalescaetano100@gmail.com (T.C.d.O.); mariangelabf11@gmail.com (M.B.F.); 2 Plants Stress Study Laboratory, University of São Paulo, Luiz de QueirózAgriculture School, P.O. Box 9, 13418-900 Piracicaba, SP, Brazil; fabiabarbosa@usp.br; 3 Biodiversity Laboratory, Minas South Federal Institute of Science and Technology—Campus Poços de Caldas, 37713-100 Poços de Caldas, MG, Brazil; elidio.monteiro@alunos.ifsuldeminas.edu.br; 4 Ecophysiology and Plant Productivity Laboratory, Goiano Federal Institute of Science and Technology—Campus Rio Verde, P.O. Box 66, 75901-970 Rio Verde, GO, Brazil; cmuller@vicosa.ufv.br

**Keywords:** macronutrients, mineral nutrition, cagaita, Brazilian Cerrado

## Abstract

The production of high-quality seedlings and their use in commercial planting reduce pressure on natural areas. *Eugenia dysenterica* DC is a native fruit tree from the Brazilian Cerrado, whose nutritional requirements are still unclear. This study aimed to evaluate the effects of nitrogen (N) and phosphorus (P) supplementation on the physiology, growth and nutrient uptake, and use efficiencies of *E. dysenterica* seedlings grown in glasshouse conditions. The following rates were used in separate experiments: 0, 50, 100, 200, and 400 mg dm^−3^ N and 0, 100, 200, 400, and 600 mg dm^−3^ P. The experiment was conducted in a randomized block with four replications. The lowest N rate (50 mg dm^−3^) increased the stomatal conductance (g_S_) and, consequently, resulted in the highest transpiration (*E*), electron transport (ETR), and photosynthetic (*A*) rates. Also, rates of 50 mg dm^−3^ and 100 mg dm^−3^ N increased the Root Uptake Efficiency (RUE) and plant Nutrient Use Efficiency (NUE) for macronutrients and the RUE for micronutrients, stimulating plant growth. Phosphorous fertilization resulted in the maximum values for photosynthesis, electron transport rate, total dry mass, and NUE at the 200 mg dm^−3^ rate. The results of this study suggest that fertilization with 50 mg dm^−3^ N and 200 mg dm^−3^ P is suitable for the development of *E. dysenterica* seedlings.

## 1. Introduction

The Cerrado is a Brazilian savanna ecosystem that has a high diversity of flora and fauna and originally covered 200 million hectares; in recent years, more than 50% of its area has been converted to grain crops and pastures [1]. *Eugenia dysenterica* DC, popularly known as cagaita, is a native fruit of the Brazilian Cerradoin the Myrtaceae family. It is an economically important species because its fruits can be used as food, having high levels of vitamin A and C [2], and in the pharmaceutical and cosmetics industries [3]; the trees are used to supply cork and timber for the construction industry as well as for firewood [4]. 

According to the regulations in the Brazilian Forestry Code (Law No. 12651 of 25 May 2012), the collection of non-timber forest products in legal reserve areas is permitted only for the purpose of subsistence farming. The law reduces the commercial exploitation of native plants because of threats to biodiversity and the number of conserved areas in the Cerrado biome. Thus, as an alternative, seedling production in orchards and their planting for commercial purposes will reduce native area exploitation. To accomplish this, more information is needed, such as the nutritional requirements of the Cerrado´s native species.

Several arboreal species of the Brazilian savanna, including cagaita, are well adapted to low-fertility soils, particularly those with high acidity, high aluminum content [5], and water deficit conditions in some months of the year; these are all typical characteristics of the Cerrado region [6]. However, studies have shown variations in the productivity of *E*. *dysenterica* [7], such as reduced fruit yield after the first five years under natural conditions [8]. Among the primary factors affecting fruit development, inconsistent production was highly correlated with low nutritional levels in the leaves. This suggests that fertilization is an important factor in the production of *E*. *dysenterica* fruits.

Appropriate nutrition allows adequate growth and increases the resistance of plants to attacks from pathogens [9]; it also contributes to the allocation of photoassimilatesto fruit development and the standardization of fruit production. Therefore, studies on the nutritional requirements of this species are important to stimulate faster and more uniform plant growth. Considering this need, it is important to highlight that nitrogen (N) and phosphorus (P) are macronutrients required in large quantities by plants during their initial development [10]. These nutrients are often present in limiting amounts in Cerrado soils. Nitrogen availability is often correlated with soil organic matter concentrations [11]. Cerrado soils tend to have minimal organic matter and, thus, low natural plant available N [6]. Plant P availability is strongly correlated with soil pH and mineralogy (including P quality) [12]. The natural P availability for plants tends to be low in these soils due to the presence of iron and aluminum oxides in the clay fraction and the low activity of clay minerals such as kaolinite [6].

Based on this background, the objective of the present study was to determine adequate rates of N and P for *E*. *dysenterica* DC seedling production, based on the physiological and morphological traits and nutrient uptake and use efficiencies of seedlings produced in Cerrado soil in glasshouse conditions.

## 2. Results

### 2.1. Physiological Traits

**Figure 1:**
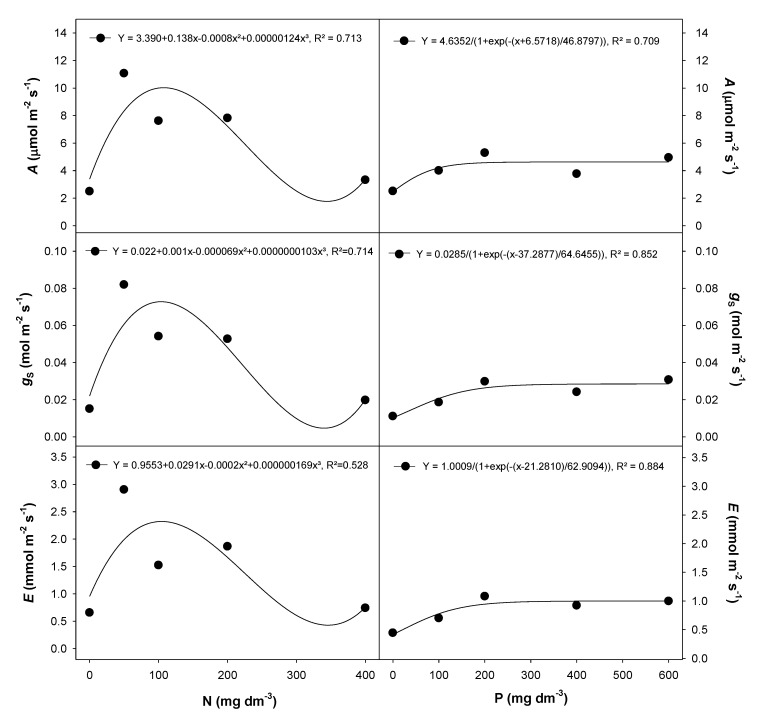
Gas exchange traits in *E. dysenterica* seedlings grown at varying rates of nitrogen and phosphorus. Photosynthetic rate (*A*), stomatal conductance (g_S_) and transpiration rate (*E*), in *Eugenia dysenterica* DC seedlings grown at varying rates of nitrogen (N) and phosphorus (P) for 278 d. Dots represent mean (*n* = 4). Means and SEM are shown as Supplementary Material (Table S1).

The N (0, 50, 100, 200 and 400 mg dm^−3^) and P (0, 100, 200, 400 and 600 mg dm^−3^) rates used in this study differentially influenced gas exchange and chlorophyll *a* fluorescence traits in *E. dysenterica* seedlings. The photosynthetic rate (*A*), stomatal conductance (g_S_) and transpiration (*E*) were lowest in plants grown in the absence of N or at the highest N rate (400 mg dm^−3^) (Figure 1). Each of these measured parameters showed a dramatic initial increase with the first increment of N fertilizer (50 mg dm^−3^) and then dropped at the highest rate (400 mg dm^−3^). Similarly, the P response increased with the first increment of fertilizer (100 mg dm^−3^ P); however, it differed as the response plateaued with increasing P rates (Figure 1).

**Figure 2:**
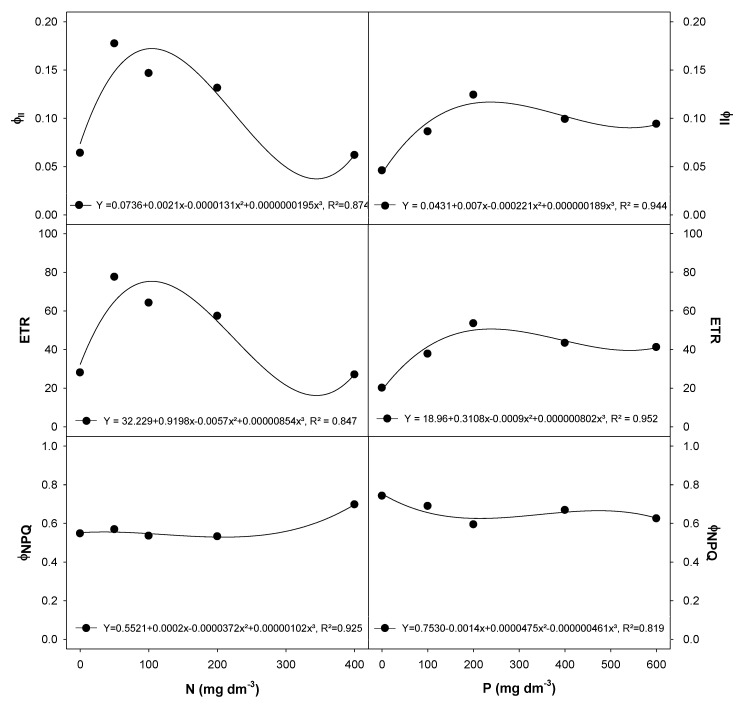
Chlorophyll *a* fluorescence traits in *E. dysenterica* seedlings grown at varying rates of nitrogen and phosphorus. Effective quantum yield of photosystem II (Φ_PSII_), electron transport rate (ETR) and regulated quantum yield of nonphotochemical energy dissipation (Φ_NPQ_) in *Eugenia dysenterica* DC seedlings grown at varying rates of nitrogen (N) and phosphorus (P) for 278 d. Dots represent mean (*n* = 4). Means and SEM are shown as Supplementary Material (Table S2).

*E. dysenterica* cultivation at various N and P rates did not change the initial chlorophyll fluorescence (F_0_, ~306.2) or the potential quantum yield of PSII (F_v_/F_m_, ~0.844) (data shown as supplementary material; Table S2). The effective quantum yield of PSII (Φ_PSII_) and the electron transport rate (ETR) were higher in plants grown with between 50 to 200 mg dm^−3^ N and 200 mg dm^−3^ P (Figure 2). The Φ_NPQ_ showed a dramatic increase in *E. dysenterica* plants exposed to 400 mg dm^−3^ N and was reduced in plants exposed to 600 mg dm^−3^ P (Figure 2). 

### 2.2. Morphological Traits 

**Figure 3:**
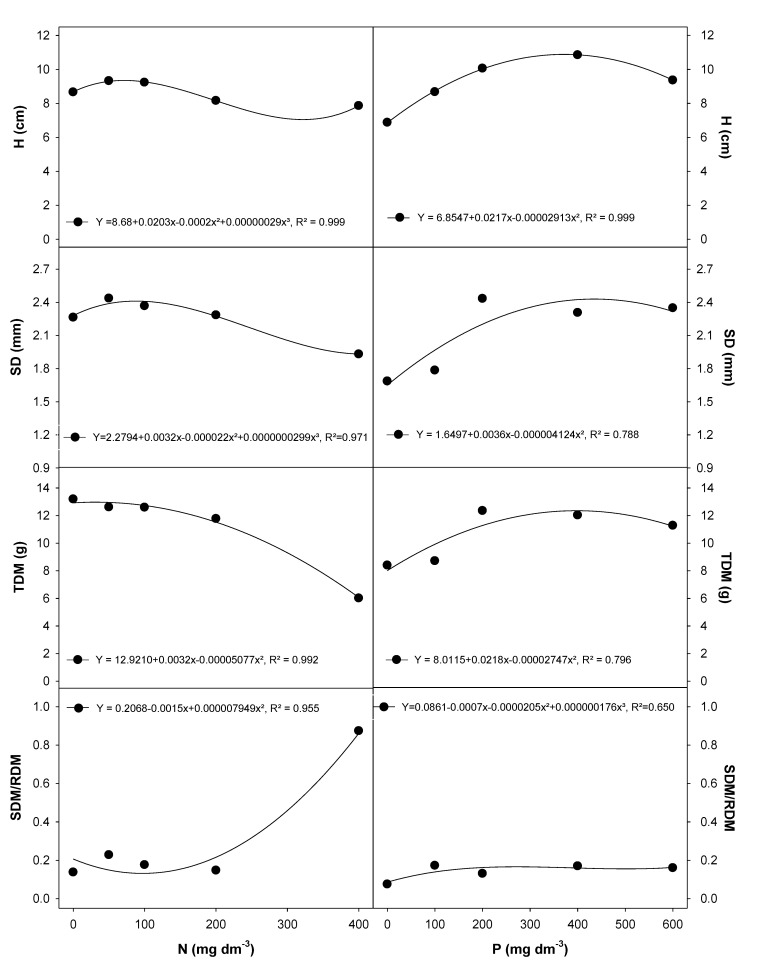
Morphological traits in *E. dysenterica* seedlings grown at varying rates of nitrogen and phosphorus. Height (H), stem diameter (SD), total dry matter (TDM), and the ratio between shoot dry matter and root dry matter (SDM/RDM) in *Eugenia dysenterica* DC seedlings grown at varying rates of nitrogen (N) and phosphorus (P) for 278 d. Dots represent mean (*n* = 4). Means and SEM are shown as Supplementary Material (Table S3).

The highest values for height (H) and stem diameter (SD) (Figure 3), and leaf number and root length (data shown as Supplementary Material; Table S3) were observed at 50 to 100 mg dm^−3^ N. The peak values were 6.6 LN and 70.8 cm RL. However, N application at higher rates (>200 mg dm^−3^) negatively affected the H and SD in *E. dysenterica* (Figure 3). The total dry matter (TDM) in plants grown at 200 and 400 mg dm^−3^ was 7% and 52% lower, respectively, than that in the 50 mg dm^−3^ N treatment (data shown as Supplementary Material; Table S3). The ratio between shoot dry matter (SDM) and root dry matter (RDM) (SDM/RDM) increased only at the highest N rate (Figure 3). 

P at 200 mg dm^−3^ produced higher values of H (Figure 3), whereas the highest values of SD, TDM and SDM/RDM in *E. dysenterica* seedlings were observed at P rates between 200 and 600 mg dm^−3^ (Figure 3). 

### 2.3. Contribution of Nitrogen and Phosphorous Rates to Physiological and Morphological Changes in Eugenia dysenterica Seedlings

**Figure 4:**
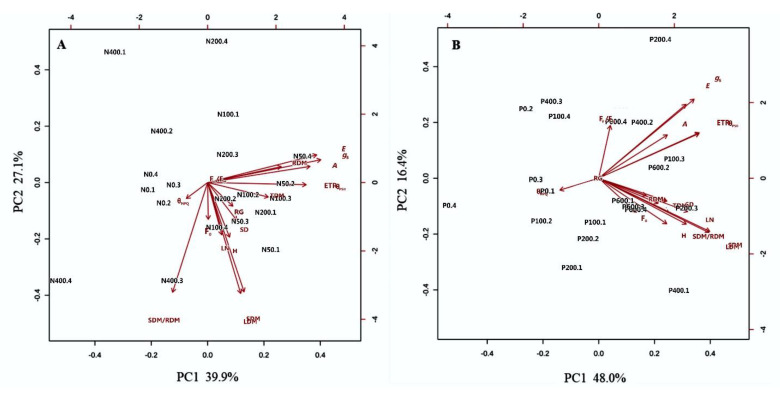
Principal component analysis (PCA) of the physiological and morphological parameters of *E. dysenterica* at varying nitrogen and phosphorus rates. Principal component analysis (PCA) of the mean values for the physiological and morphological characteristics of *Eugenia dysenterica* DC grown at varying (**A**) nitrogen (N; 0, 50, 100, 200, and 400 mg dm^−3^) and (**B**) phosphorus (P; 0, 100, 200, 400, and 600 mg dm^−3^) rates for 278 d. Images A and B show score variations in these traits along the first two main component axes, with the percentage of variation explained.

The contributions of N and P doses to physiological and morphological traits were determined by principal component analysis (PCA). The first two main components (PC1 and PC2) explained 67.0% and 64.4% of the total data variation for the N and P rates, respectively (Figure 4). It was observed that the variables *A,* g_S_*, E,* Φ_PSII_, ETR, RDM, TDM and SDM madea higher contribution toPC1 (Figure 4A). At the rate of 50 mg dm^−3^ N, *E. dysenterica* seedlings presented higher values of **A, g_S_, *E*, Φ_PSII_, ETR and SDM than in other treatments (Figure 4A), and the physiological variables showed higher values than the morphological variables.

Phosphorus positively influenced all physiological and morphological traits of *E. dysenterica* (Figure 4B). However, it was observed that the 200 mg dm^−3^ rate of P resulted in the highest values of *A,* g_S_, *E*, Φ_PSII_ and ETR. The PC1 also indicated that LN, SDM/RDM, and SDM were highly correlated with 200 mg dm^−3^ P.

### 2.4. Nitrogen and Phosphorous Uptake and Use Efficiency

**Figure 5:**
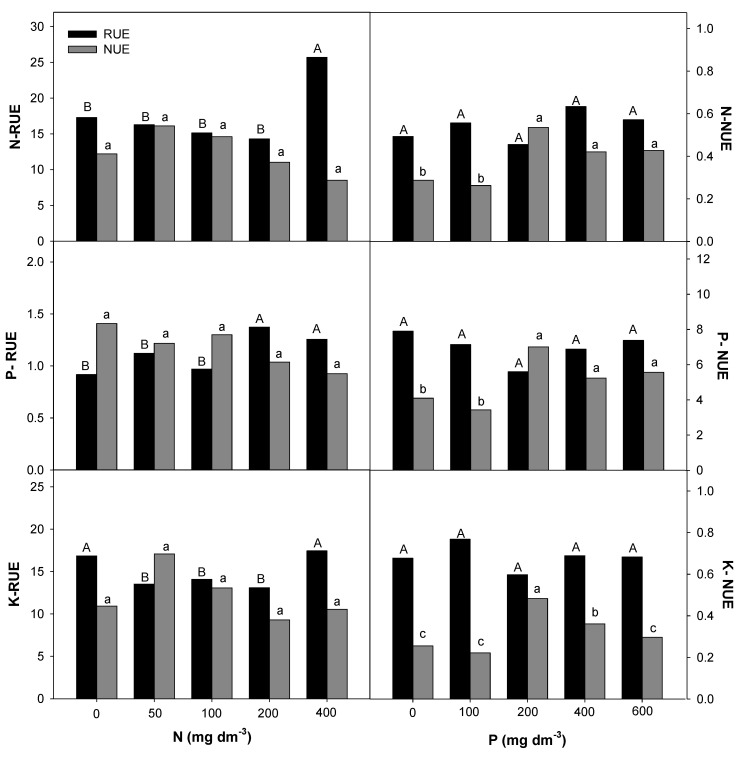
Root uptake efficiency (RUE) and nutrient use efficiency (NUE) for nitrogen (N), phosphorus (P), and potassium (K) in *E. dysenterica* seedlings. Root uptake efficiency (RUE, mg g^−1^) and plant nutrient use efficiency (NUE, g^2^ mg^−1^) for the macronutrients N, P, and K in *Eugenia dysenterica* DC seedlings grown at varying rates of nitrogen and phosphorus for 278 d. Bars represent means (*n* = 4). Means followed by the same letters, uppercase for RUE and lowercase for NUE, do not differ by the Scott-Knott clustering test (*p* > 0.05).

**Figure 6:**
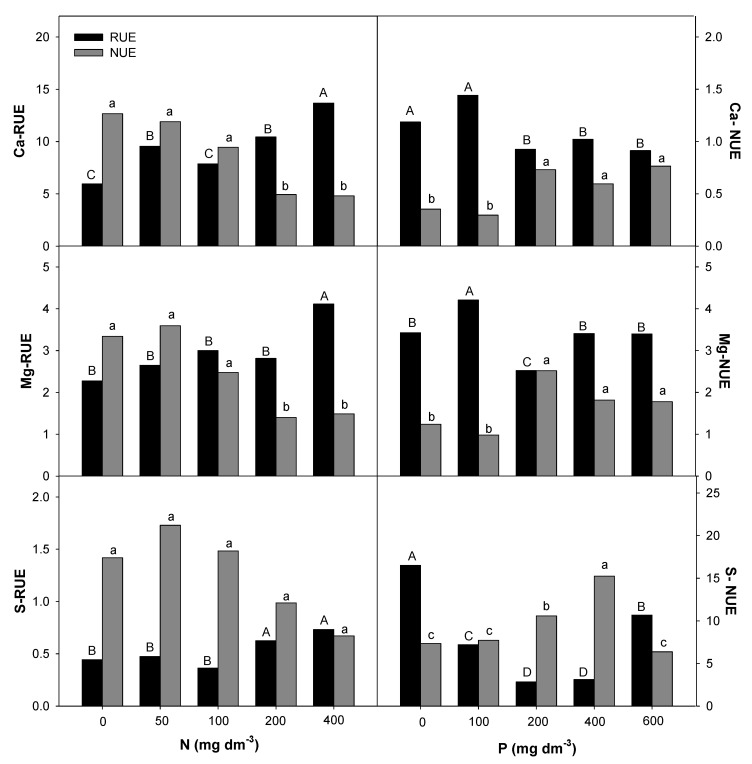
Root uptake efficiency (RUE) and nutrient use efficiency (NUE) for calcium (Ca), magnesium (Mg), and sulfur (S) in *E. dysenterica* seedlings. Root uptake efficiency (RUE, mg g^−1^) and plant nutrient use efficiency (NUE, g^2^ mg^−1^) for the macronutrients Ca, Mg, and S in *Eugenia dysenterica* DC seedlings grown at varying rates of nitrogen and phosphorus for 278 d. Bars represent means (*n* = 4). Means followed by the same letters, uppercase for RUE and lowercase for NUE, do not differ by the Scott-Knott clustering test (*p* > 0.05).

**Figure 7:**
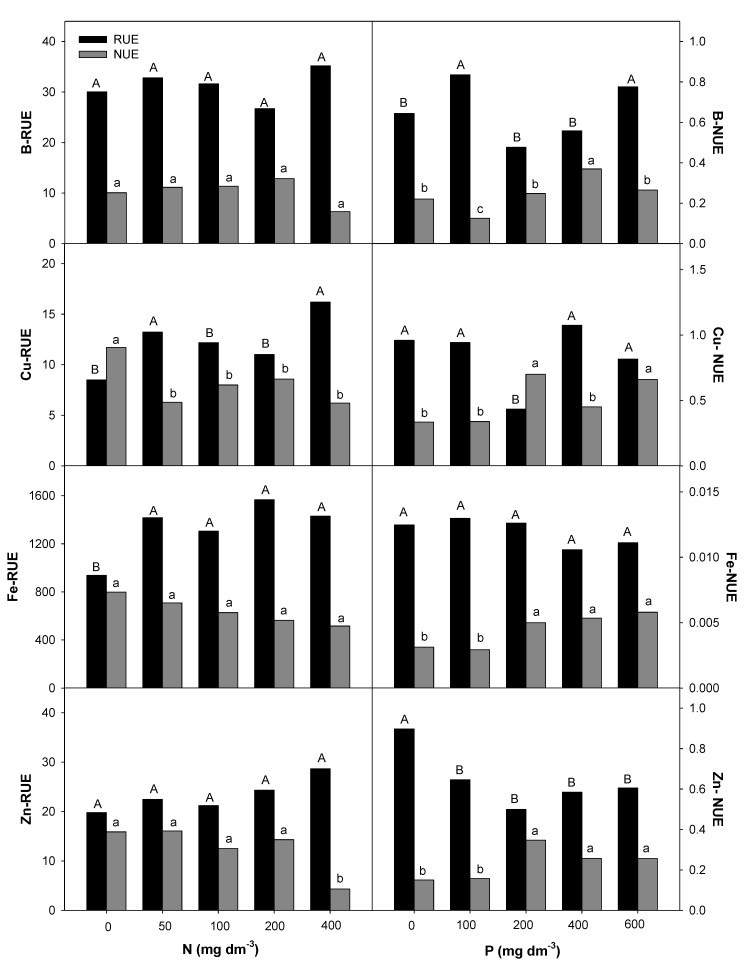
Root uptake efficiency (RUE) and nutrient use efficiency (NUE) for micronutrients in *E. dysenterica* seedlings. Root uptake efficiency (RUE, mg g^−1^) and plant nutrient use efficiency (NUE, g^2^ g^−1^) for the micronutrients boron (B), copper (Cu), iron (Fe), and zinc (Zn) in *Eugenia dysenterica* DC seedlings grown at varying rates of nitrogen and phosphorus for 278 d. Bars represent means (*n* = 4). Means followed by the same letters, uppercase for RUE and lowercase for NUE, do not differ by the Scott-Knott clustering test (*p* > 0.05).

The Root Uptake Efficiency (RUE) and plant Nutrient Use Efficiency (NUE) for macro- and micronutrients in *E. dysenterica* seedlings were differentially affected by the availability of N and P in the soil (Figure 5, Figure 6 and Figure 7). The increase in the N rate positively affected the RUE-N and RUE-Mg at the highest rate. RUE-P and RUE-S were stimulated from 200 mg dm^−3^ N, and RUE-Ca, RUE-Cu, and RUE-Fe were stimulated from the lowest rate (50 mg dm^−3^ N). Even less responsive, NUE-Ca and NUE-Mg decreased from 200 mg dm^−3^ N, and NUE-Cu and NUE-Zn were reduced at all doses. The NUE of the other macronutrients was not affected by the increasing N rate.

P availability positively affected RUE-Mg and RUE-B at the lowest rate (100 mg dm^−3^) in *E. dysenterica* plants. RUE-Ca was reduced starting from 100 mg dm^−3^ P, and RUE-S and RUE-Zn were reduced at all P rates. The uptake efficiencies of the other nutrients evaluated in *E. dysenterica* plants were not affected by the varying doses of P in the soil (Figure 6). The NUE values for most nutrients (N, P, K, Ca, Mg, S, Cu, Fe, Zn) were higher at 200 mg dm^−3^ P, that at other rates but did not differ significantly between the higher rates (400 and 600 mg dm^−3^ P) (Figure 5, Figure 6 and Figure 7).

## 3. Discussion

### 3.1. Nitrogen Fertilization Increased Photosynthetic Rates of Eugenia dysenterica

The N and P play key roles in the photochemical and biochemical stages of photosynthesis because they are constituents of photosynthetic process-related proteins, including photosynthetic pigments [13] and the enzyme RuBisCO [14]. Changes in RuBisCO activity can occur either because of changes in the levels of P in the plant that affect the allocation of N to RuBisCO [15] or because N deficiency can hinder the uptake of P and induce changes in RuBisCO activity [14]. The availability of N in the soil contributed to the higher photosynthetic activity in the *E. dysenterica* seedlings. However, we observed that the highest values for the photosynthetic rate and photochemical processes occurred in plants that were cultivated at the lowest rate of N (50 mg dm^−3^), demonstrating the low requirement for this nutrient in the early stages of *E. dysenterica* development.

This trend was also observed in *Passiflora alata* seedlings that obtained maximum photosynthetic values at low N doses (up to 146 mg kg^−1^) 176 d after staking [16]. It has been reported that tree plants exhibit increased N demand with increasing age and during fruit production [17], and two main sources of N that fruit trees use for their vegetative growth and reproduction are root N uptake and internal N cycling. The N available for root uptake can be derived from mineral fertilizers or from the mineralization of natural soil N. Plant roots are able to take up the available N forms, i.e., nitrate (NO_3_^−^) and ammonium (NH_4_^+^) [11,18]. 

The rates of P used in the present study significantly altered the photosynthetic activity of *E. dysenterica* seedlings. The photosynthetic rate (*A*), stomatal conductance (g_S_), transpiration rate (*E*), electron transport rate (ETR), and PSII quantum yield (Φ_PSII_) were responsive to 200 mg dm^−3^ P. The P availability can stimulate stoma production by epidermal cells, and consequently increase stomatal conductance and photosynthesis [19]. A higher stomatal density was observed by Seika and Yano [20] in *Vigna sinensis* subjected to 450 mg of P fertilizer. On the other hand, Salter et al. [21] found that native plants grown under P-limited conditions showed impaired photosynthesis due to reduced stomatal conductance. Moreover, the increase in photosynthetic rates and photochemical processes observed in *E. dysenterica* in both N and P treatments was accompanied by lower nonphotochemical energy dissipation (Φ_NPQ_), suggesting increased thermal dissipation related to the xanthophyll cycle [22] as an alternative sink for excess energy under stress conditions.

The contribution of phosphorous to the photosynthetic process is related to its function as a substrate for ATP synthesis in the chloroplast stroma, whereas inorganic P deficiency caused by low P availability reduces ATP production in the stroma and CO_2_ fixation [23]. In addition to maintaining plant development, high photosynthetic rates are expected to result in higher fruit yield and uniformity. This occurs because the yield per plant is directly related to the production of soluble solids [24] and fruits are the main sinks of photoassimilates during the reproductive stage.

### 3.2. EugeniaDysenterica Growth Was Stimulated by Phosphate Fertilization

Adequate N and P are important nutrients for the initial development of tree plants [25]. The N is required throughout plant growth, and N deficiency is considered one of the most limiting factors for plant growth [11,26]. Besides a lack of fertilization, this nutrient deficiency can also be related to the form of N absorbed by plants (mostly nitrate), which is readily leachable. To favor N acquisition and mobilization, the stimulation of root development and plant biomass production should be promoted [12,27].

P is recognized as an important component of plants as an energy carrier and in nucleic acids and signaling pathway proteins (such as protein kinases) [28]. P is the main limiting element for *Eucalyptus grandis* seedling growth [29] and is critical for the development of slow-growing tree species [30,31].

An increase in the height and stem diameter of *Cedrela fissilis* seedlings, which also occur in the Cerrado biome, was promoted by increased rates of N (40 to 160 mg dm^−3^) [32]. Nevertheless, in the present study, the increase in N fertilization up to 200 mg dm^−3^ did not affect the growth of the young *E. dysenterica* plants under glasshouse conditions. As observed for *E. dysenterica*, mature watermelon plants provided with a high N supply also showed a lower performance in root growth than plants grown under low N conditions [33]. Similarly, previous studies reported that the morphological traits of *Talisia esculenta* [25] and *E. dysenterica* [34], particularly height and stem diameter, were not responsive to N doses under controlled conditions. This suggests that this species has low nutritional requirements for N in the earlier stages of development. 

On the other hand, the cultivation of plants at the highest N rate (400 mg dm^−3^) had negative effects on the growth of young *E. dysenterica* after 278 d of cultivation. Similar behavior in growth and dry mass production was observed at doses higher than 504 mg dm^−3^ N in *Talisia esculenta* seedlings [25] and at doses of 200 mg dm^−3^ N in *Annona crassiflora* [35].

Unlike N fertilization, P fertilization from the rate of 100 mg dm^−3^ significantly influenced the height of *E. dysenterica*, while rates from 200 mg dm^−3^ P increased the stem diameter. This demonstrates the role of P in shoot biomass production in *E. dysenterica*. Similarly, *Araucaria angustifolia* was shown to be responsive to increased P availability in terms of height, stem diameter, and shoot dry matter, with higher values at 472 mg dm^−3^ P fertilization [36]. Bessa et al. [37] also observed a high correlation between plant growth traits and the availability of N, P, K, and S for *E. dysenterica* seedlings grown in hydroponic solution for 190 d.

The SDM/RDM of *E. dysenterica* was not influenced by the N rates (0 to 200 mg dm^−3^) and showed higher values in P-fertilized plants, than in the absence of added P. The higher translocation of photoassimilates correlated with auxin accumulation [38] is a characteristic behavior of Cerrado species that are well adapted to nutrient-limiting conditions [39]. An important aspect to be considered when evaluating the response of *E. dysenterica* seedlings to nutrient rates is related to the morphology and initial establishment of the species. The interleaved periods of root and shoot development observed in the growth of native plants [39] may influence the biometric and nutritional responses of the seedlings.

### 3.3. Effect of Nitrogen and Phosphorous Rates on Root Uptake and Plant Nutrient Use Efficiencies

Studies on the uptake and use efficiencies of native species are rare; however, attention has been paid to the importance of N and P in the initial development of these plants [40,41]. The interaction of N with other nutrients varies with environmental conditions (soil and climate), species, and genotype, and is affected by the forms of N absorbed by plants (N-NO_3_^−^ or N-NH_4_^+^) [42]. The N-RUE in *E. dysenterica* seedlings was responsive up to the highest evaluated rate (400 mg dm^−3^ N); however, the seedlings showed lower NUE under this condition, possibly suggesting a negative effect on the dry matter production of the species. Nevertheless, it has been reported that N redistribution in trees occurs seasonally [26], and a higher N requirement can begin after two years of cultivation [43]. 

The uptake and use efficiencies for P were affected by the gradual increase in the supply of N. This is important because soils with a high level of iron and aluminum oxides, such as Cerrado soils, preferentially present positive charges that are capable of adsorbing various anions, such as phosphate ions, making P less available to plants [12,44]. Even if it has not been observed in *E. dysenterica* seedlings, the positive interaction between N and K has been described for crops [42], because K plays an important role as a counterion to nitrate (NO_3_^−^) transport in xylem [45]. 

The N availability favored the uptake efficiency of Ca, Mg and S. The transport of nitrate ions in plasma membranes requires the activity of proton pumps (H^+^-ATP-ase) that depend on Mg as an enzyme cofactor [46]. The Ca is taken up by the root apical zone; however, it is immobile in the phloem [47]. This nutrient is required during cell division and cell wall synthesis and is therefore important for plant development [48]. Like N, S is highly mobile in soil and is taken up mainly in the form of sulfate (SO_4_^2^^−^) [47]. In plant chloroplasts, S is a component of nitrite reductase, an enzyme responsible for reducing NO_2_^−^ to NH_3_ [49], where nitrite is an intermediate product that is toxic to plants. 

Unlike for N, the availability of various P rates had little effect on the nutrient uptake efficiency of *E. dysenterica*; however, it increased the plant nutrient use efficiency, especially at 200 mg dm^−3^ P. Trees have been reported to store P in biomass as a long-term adaptive mechanism [31]. According to these authors, in slow-growing species, there is also a tendency to accumulate more P than N to partially offset the gradual decrease in P in the soil. 

The nutrient K plays an important role in stomatal control and cell expansion [50]. It has also been observed that K use efficiency is directly related to sugar accumulation and, consequently, fruit development and quality [51,52]. The higher K-NUE in *E. dysenterica* was observed at rates of 50 mg dm^−3^ N and 200 mg dm^−3^ P. These results were similar to those observed in *Stryphnodendron polyphyllum*, a native Cerrado species that showed higher NUE-K with a supply of 62 mg dm^−3^ N and 125 mg dm^−3^ P [40]. 

The interrelationship between P and Mg occurs in various metabolic processes in plants because Mg is required by the phosphorylative enzymes that incorporate and transfer inorganic P, in addition to being responsible for forming cross-bridges between these enzymes and ATP and ADP [9]. As observed in *E. dysenterica,* the highest NUE-Mg was found under 200 mg dm^−3^ P. A similar interaction was observed for the Cerrado species *Jacaranda decurrens* subsp. s*ymmetrifoliolata,* in which P addition increased the Mg concentration in the roots of these plants [53]. P is the most limiting nutrient with respect to growth and biomass production in *E. dysenterica* seedlings [34]; in the present work, 200 mg dm^−3^ P caused increases in macro- and micronutrient use efficiency. 

Like macronutrients, micronutrients, despite being required in smaller quantities, had their uptake and use efficiency affected by the availability of N and P in the soil. Cerrado soils have low availability of boron (B), copper (Cu), molybdenum (Mo), and zinc (Zn) [6], possibly compromising the development of native trees [54] and affecting fruit production. In these soils, pH correction by liming may decrease the availability of micronutrients such as Mn, Cu, and Zn [3,55], highlighting the importance of understanding micronutrient uptake and use efficiencies for *E. dysenterica* seedling production.

The availability of N favored Cu, Fe, and Zn uptake in *E. dysenterica* seedlings. The interactions of N with micronutrients occur because of changes in rhizosphere pH values and the N forms used. N available as nitrate (NO_3_^−^) causes increased rhizosphere pH, with a reduction in micronutrient uptake, while ammonium N (NH_4_^+^) results in decreased rhizosphere pH values and consequently higher micronutrient uptake [42]. Therefore, the use of ammoniacal sources may favor the uptake of micronutrients in *E. dysenterica*. Similarly, Carlisle et al. [56] found that the supply of ammoniacal N resulted in increased Fe, Cu, and Zn concentrations in wheat plants. Even at higher uptake efficiencies, the use efficiency of these nutrients was lower in *E. dysenterica* seedlings, under a high soil N rate (600 mg dm^−3^).

The RUE-B was not affected by the varying rates of N and increased at 100 mg dm^−3^ in soil. Liming generally decreases B because the formation of Al(OH)_3_ by Al^3^^+^ precipitation in solution allows the uptake of large amounts of B [6]. The NUE-B for *E. dysenterica* seedlings was lower with N availability and greater with the supply of 400 mg dm^−3^ P. B has low mobility in plants but is essential because it influences Ca^2^^+^ utilization and cell membrane integrity [57]; this is because it is associated with the pectin content in the cell wall [58].

The highest RUE-Cu was observed at the lowest N rate (50 mg dm^−3^) but higher NUE-Cu was observed when the plants were exposed to increasing P rates. This emphasizes the importance of phosphate fertilization for micronutrient use efficiency in *E. dysenterica* seedlings. Exchangeable Cu is adsorbed by organic matter. In the Cerrado scenario, with low organic matter content and low pH, exchangeable Cu is expected to be prevalent and available to plants, as observed in the appropriate Cu levels in various tree species [59]. Cu is a constituent of important oxidase enzymes, including cytochrome oxidase, ascorbic acid oxidase, and lactase, and is involved in the photosynthetic process [42].

The highest NUE-Zn was observed at 200 mg P dm^−3^ in *E. dysenterica* seedlings. Increasing P rates also provided higher NUE-Zn in *Stryphnodendron polyphyllum* seedlings [40]. These data suggest that micronutrient retranslocation depends on an adequate supply of P, which promotes uniform growth and favors fruit production. 

We observed favorable responses in the physiological and morphological traits and nutrient use efficiency of *E. dysenterica* seedlings grown under 200 mg dm^−3^ P and 50 mg dm^−3^ N, with P being more limiting to the initial development of this species. Multivariate analysis allowed us to verify that both physiological and morphological assessments were important in the investigation. Our data also demonstrate the importance of fertilization in seedling production programs for commercial plantations, the consolidation of seed orchards and even the use of these species in restoration programs in degraded areas. It is important to favor more uniform fruit production, as non-uniform fruit is a common problem under natural conditions in the Cerrado [7].

## 4. Materials and Methods 

### 4.1. Plant Material and Growth Conditions

Ripe *E. dysenterica* fruits were collected from plants located in the municipality of Montes Claros de Goiás, GO, Brazil (19°53′ S latitude, 44°25′ W longitude and 592 m altitude). The specimens were deposited in the herbarium of the Goiano Federal Institute (IF Goiano), Campus Rio Verde (n. 630/2017). After collection, the fruits were homogenized and processed to obtain the seeds.

For both studies, a clayey red Latosol was collected from the 20–40 cm soil layer in a native area of the IF Goiano. The soil had the following chemical attributes, determined according to Silva et al. [60]: pH (CaCl_2_) = 4.3; organic matter = 27 g kg^−1^; P (Mehlich-1) = 1.0 mg dm^−3^; K = 0.11 cmol_c_ dm^−3^; Ca = 0.4 cmol_c_ dm^−3^; Mg = 0.1 cmol_c_ dm^−3^; Al = 0.3 cmol_c_ dm^−3^; H + Al = 4.5 cmol_c_ dm^−3^; aluminum saturation = 33% and base saturation (V) = 12%.Based on these values, the soil was incubated (for 20 d) with dolomitic limestone (PRNT 70%), adopting the basis saturation criterion (V%) to 60%.

The experiments were carried out in pots containing 2.8 dm^3^ of the soil, which was previously subjected to basic fertilization [61] with 100 mg dm^−3^ potassium (K), 40 mg dm^−3^ sulfur (S), 1.33 mg dm^−3^ copper (Cu), 0.81 mg dm^−3^ boron (B), 4 mg dm^−3^ zinc (Zn), 3 mg dm^−3^ manganese (Mn), and 0.15 mg dm^−3^ molybdenum (Mo). Calcium (Ca) and magnesium (Mg) were supplied by liming. The salts used in the fertilizer solutions were KCl, MnSO_4_, ZnSO_4_, CuSO_4_, H_3_BO_3_, and MoO_3_. After fertilization, four cagaita seeds were added per pot. At 30 d after germination, thinning was performed, retaining two plants per pot. The criteria for plant selection involved uniformity of morphological features, i.e., two expanded pairs of leaves, the absence of contamination (fungi), and adequate spacing in the pot. During the experimental period, the soil moisture was checked every two days and maintained well irrigated with deionized water when necessary.

**Figure 8:**
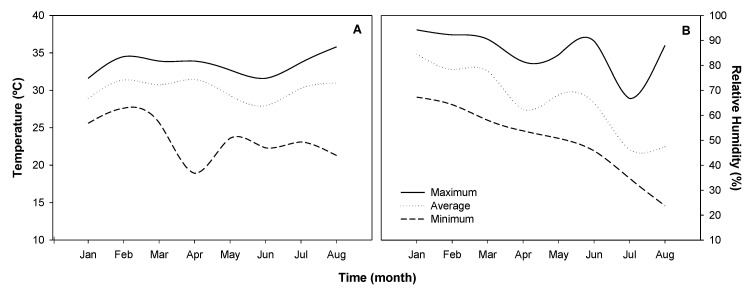
Glasshouse temperature (°C) and relative humidity (%) conditions during the experimental period. Temperature (°C) maximum, medium, and minimum (**A**); and relative humidity (%) maximum, medium, and minimum (**B**) in the glasshouse during the experimental period.

The experiments were carried out in a glasshouse of the Plant Tissue Culture Laboratory of IF Goiano, Rio Verde, Goiás, Brazil. Temperature and humidity conditions during the experimental period were recorded using a data logger (Novus, model LogBox-RHT-LCD, Porto Alegre, RS, Brazil) and are presented in Figure 8.

### 4.2. Treatments and Experimental Design

Two independent experiments were conducted, one for each nutrient evaluated. For the treatments corresponding to rates of N and P, the nutrients were not added to the basic fertilizer previously mentioned.

The experimental design was completely randomized, with five dosages for each nutrient (N and P) and four replicates, with 20 pots per experiment. The N and P rates in the treatments were added separately, using the salts NH_4_SO_4_, NH_4_Cl, NH_4_(H_2_PO_4_), and CaHPO_4_, corresponding to: (i) N: 0, 50, 100, 200, and 400 mg dm^−3^; and (ii) P: 0, 100, 200, 400, and 600 mg dm^−3^. 

### 4.3. Evaluations

The experiments lasted 278 d. Then, the physiological and morphological traits were measured, and samples were collected to analyze their nutritional content.

#### 4.3.1. Physiological Traits 

Gas exchangeparameters were evaluated in the last completely expanded leaves to measure the photosynthetic rate (*A*, µmol m^−2^ s^−1^), stomatal conductance (g_S_, mol m^−2^ s^−1^) and transpiration rate (*E*, mmol m^−2^ s^−1^). The evaluations were carried out between 9:00 and 11:00 am using an Infrared Gas Analyzer (IRGA; LI-6400XT, Li-Cor, Lincoln, NE, USA), under constant photosynthetically active radiation (PAR, 1000 μmol of photons m^−2^ s^−1^) and the atmospheric concentration of CO_2_ (~405 μmol mol^−1^), temperature (~31 °C) and relative humidity (~36%).

The characteristics of chlorophyll *a* fluorescence were measured using a fluorometer (6400-40, Li-Cor, Lincoln, NE, USA) coupled with an IRGA on the same leaf for which the gas exchange data were obtained. Initially, the leaves were adapted to the dark (when the PSII reaction centers are open) to obtain the minimum (F_0_) and maximum (F_m_) fluorescence, with the application of the measuring light (~0.03 µmol m^−2^ s^−1^) and a saturation pulse (>3000 µmol m^−2^ s^−1^), respectively. The potential quantum yield of photosystem II (PSII) was determined as F_v_/F_m_ = (F_m_ − F_0_)/F_m_. After illumination with continuous actinic light (~1000 µmol m^−2^ s^−1^) for 40 s, a saturation pulse was applied to determine the maximum fluorescence (F_m_') and steady-state (F_s_) in the leaves adapted to light. The effective quantum yield of photochemical energy conversion in PSII (Φ_PSII_ = [F_m_' − F_s_]/F_m_') and the quantum yields of regulated nonphotochemical energy dissipation (Φ_NPQ_ = [F_s_/F_m_'] − [F_s_/F_m_]) were calculated.

The Φ_PSII_ was used to estimate the apparent rate of electron transport (ETR = Φ_PSII_ × PAR × ABS_leaf_ × 0.5; μmol electrons m^−2^ s^−1^), in which PAR is the photosynthetically active radiation (µmol photons m^−2^ s^−1^); ABS_leaf_ corresponds to the fraction of incident light that is absorbed by the leaves, and 0.5 is the value corresponding to the fraction of excitation energy distributed to PSII.

#### 4.3.2. Morphological Traits

The plants were measured to determine their height (H, cm), stem diameter (SD, mm), leaf number (LN), and root length (RL, cm). The shoot and root system were collected and dried individually in a forced-air oven at 65 °C until reaching a constant weight to obtain the shoot dry matter (SDM, g) and root dry matter (RDM, g). From these values, the ratio of SDM/RDM was calculated.

#### 4.3.3. Root Uptake Efficiency and Plant Nutrient Use Efficiency

For the determination of the nutritional content of the leaves and roots, the dry plant material was ground in a Wiley mill, and ~500 mg of the dry ash was extracted with nitric-percholoric (3:1) digestion according to Silva et al. [60]. Nitrogen (N) was measured via titration by the Kjeldahl method using a nitrogen distiller (TE-0364, Tecnal, Piracicaba, SP, Brazil). Phosphorous (P) and sulfur (S) were determined via the molybdenum blue and turbidity with barium chloride methodologies, respectively, using molecular absorption spectrophotometry (SP1105, Tecnal, Piracicaba, SP, Brazil). Potassium (K) was analyzed using flame photometry (B462, Tecnal, Piracicaba, SP, Brazil), and Ca, Mg, Cu, Fe, and Zn were analyzed using atomic absorption spectrophotometry (Savant AA, GBC Scientific Equipment, Braeside, VIC, Australia). B was analyzed using the azomethine-H method [57]. The nutrient contents were expressed as g kg^−1^. 

Based on these findings, the nutrients were determined on a dry matter basis (in g plant^−1^) and multiplied by the nutrient content (in g and mg for macronutrients and micronutrients, respectively). Using the results of the dry matter and nutrient contents in the plant, the root uptake efficiency (RUE) and plant nutrient use efficiency (NUE) indexes were calculated according to Swiader et al. [62] and Siddiqi and Glass [63], respectively, using the following equations:

$$RUE=\frac{Nutrient\;Content\;\left(shoot\;and\;root\;\right)\;\left(mg\right)}{Root\;Dry\;Matter\;\left(g\right)}$$

$$NUE=\frac{{\left(Total\;Leaf\;Dry\;Matter\left(g\right)\right)}^{2}}{Nutrient\;Content\;\left(mg\right)}$$

### 4.4. Statistical Analysis

Physiological and morphological data were submitted to analysis of variance (ANOVA) by the F test (*p* ≤ 0.05) and adjusted to linear or nonlinear regression models. Standardized data were also assessed by principal component analysis (PCA). The root uptake efficiency and plant nutrient use efficiency data were analyzed by ANOVA and the means were grouped using the clustering Scott-Knott method (*p* ≤ 0.05). All statistical analyses were performed using the R program (v. 3.6.1, R Foundation, Vienna, Austria), and the graphs were plotted using SigmaPlot software (v. 11.0, Systat Software GmbH, Erkrath, Germany).

## 5. Conclusions

Nitrogen fertilization increased the photosynthetic activity and growth of *E. dysenterica* seedlings at rates between 50 and 200 mg dm^−3^. The highest evaluated N rate (400 mg dm^−3^) resulted in the lowest nutrient use efficiency.

Phosphate fertilization from 100 mg dm^−3^ increased the growth of *E. dysenterica*. The highest photosynthetic activity and macro- and micronutrient use efficiencies were observed at the P rate of 200 mg dm^−3^. Taken together, the data suggest that fertilization with 50 mg dm^−3^ N and 200 mg dm^−3^ P is suitable for the development of *E. dysenterica* seedlings.

## Supplementary Materials

The following are available online at https://www.mdpi.com/2223-7747/9/6/722/s1, Table S1: Photosynthetic rate (*A*, µmol m^−2^ s^−1^), stomatal conductance (g_S_, mol m^−2^ s^−1^) and transpiration rate (*E*, mmol m^−2^ s^−1^) in *Eugenia dysenterica* DC seedlings grown at varying rates of nitrogen (N, mg dm^−3^) and phosphorus (P, mg dm^−3^) for 278 d. Table S2: Initial fluorescence (F_0_), potential quantum yield of photosystem II (F_v_/F_m_), electron transport rate (ETR), effective quantum yield of photosystem II (Φ_PSII_) and regulated quantum yield of nonphotochemical energy dissipation (Φ_NPQ_) in *Eugenia dysenterica* DC seedlings grown at varying rates of nitrogen (N, mg dm^−3^) and phosphorus (P, mg dm^−3^) for 278 d. Table S3: Height (H, cm), stem diameter (SD, mm), leaf number (LN), root length (RL, cm), total dry mass (TDM, g) and the ratio between shoot dry matter and root dry matter (SDM/RDM) in *Eugenia dysenterica* DC seedlings grown at varying rates of nitrogen (N, mg dm^−3^) and phosphorus (P, mg dm^−3^) for 278 d.
