# Supplementary material for: Growth, Physiology and Nutrient Use Efficiency in Eugenia dysenterica DC under Varying Rates of Nitrogen and Phosphorus
Source: Plants (Basel). 2020 Jun 8;9(6):722. doi: 10.3390/plants9060722 (PMC7355562; doi:10.3390/plants9060722)
Supplement: Supplementary file 1 [file plants-09-00722-s001.zip › Supplementary Material S1.docx]

**Table S1.** Photosynthetic rate (*A*, µmol m^−2^ s^−1^), stomatal conductance (*g*_S_, mol m^−2^ s^−1^) and transpiration rate (*E*, mmol m^−2^ s^−1^) in *Eugenia dysenterica* DC seedlings grown at varying rates of nitrogen (N, mg dm^−3^) and phosphorus (P, mg dm^−3^) for 278 d.

|  | | ***A*** | ***g*_S_** | ***E*** |
| --- | --- | --- | --- | --- |
|  | 0 | 2.50 ± 0.12c | 0.015 ± 0.002c | 0.66 ± 0.08c |
|  | 50 | 11.06 ± 1.70a | 0.082 ± 0.016a | 2.90 ± 0.56a |
| N | 100 | 7.61 ± 1.14b | 0.054 ± 0.008b | 1.52 ± 0.29b |
|  | 200 | 7.82 ± 1.15b | 0.053 ± 0.007b | 1.87 ± 0.11b |
|  | 400 | 3.33 ± 0.66c | 0.020 ± 0.003c | 0.74 ± 0.14c |
|  | 0 | 2.51 ± 0.41a | 0.011 ± 0.002a | 0.44 ± 0.08a |
|  | 100 | 4.01 ± 1.01a | 0.019 ± 0.005a | 0.70 ± 0.17a |
| P | 200 | 5.30 ± 1.78a | 0.030 ± 0.014a | 1.08 ± 0.53a |
|  | 400 | 3.77 ± 0.46a | 0.024 ± 0.004a | 0.92 ± 0.21a |
|  | 600 | 4.95 ± 0.85a | 0.031 ± 0.005a | 1.00 ± 0.13a |

Data represent mean ± SEM (*n* = 4). Means followed by the same letters at column do not differ by the Scott-Knott clustering test (*p* > 0.05).
