# Supplementary material for: Growth, Physiology and Nutrient Use Efficiency in Eugenia dysenterica DC under Varying Rates of Nitrogen and Phosphorus
Source: Plants (Basel). 2020 Jun 8;9(6):722. doi: 10.3390/plants9060722 (PMC7355562; doi:10.3390/plants9060722)
Supplement: Supplementary file 1 [file plants-09-00722-s001.zip › Supplementary Material S2.docx]

**Table S2.** Initial fluorescence (F_0_), potential quantum yield of photosystem II (F_v_/F_m_), electron transport rate (ETR), effective quantum yield of photosystem II (Φ_PSII_) and regulated quantum yield of nonphotochemical energy dissipation (Φ_NPQ_) in *Eugenia dysenterica* DC seedlings grown at varying rates of nitrogen (N, mg dm^−3^) and phosphorus (P, mg dm^−3^) for 278 d.

|  | | **F_0_** | **F_v_/F_m_** | **ETR** | **Φ_PSII_** | **Φ_NPQ_** |
| --- | --- | --- | --- | --- | --- | --- |
|  | 0 | 256.1 ± 98.8a | 0.87 ± 0.050a | 28.1 ± 2.2b | 0.064 ± 0.005b | 0.55 ± 0.030b |
|  | 50 | 399.8 ± 11.2a | 0.85 ± 0.005a | 77.6 ± 8.2a | 0.177 ± 0.019a | 0.57 ± 0.010b |
| N | 100 | 326.6 ± 125.4a | 0.88 ± 0.046a | 64.2 ± 3.9a | 0.147 ± 0.009a | 0.54 ± 0.051b |
|  | 200 | 366.0 ± 108.3a | 0.85 ± 0.030a | 57.4 ± 7.3a | 0.131 ± 0.017a | 0.53 ± 0.058b |
|  | 400 | 364.0 ± 20.3a | 0.84 ± 0.018a | 27.1 ± 4.7b | 0.062 ± 0.011b | 0.70 ± 0.019a |
|  | 0 | 192.0 ± 57.6a | 0.90 ± 0.029a | 20.1 ± 5.4b | 0.046 ± 0.012b | 0.74 ± 0.027a |
|  | 100 | 264.1 ± 109.9a | 0.90 ± 0.044a | 37.8 ± 9.7b | 0.086 ± 0.022b | 0.69 ± 0.047a |
| P | 200 | 235.1 ± 39.6a | 0.62 ± 0.203a | 53.5 ± 11.2a | 0.124 ± 0.026a | 0.59 ± 0.047b |
|  | 400 | 293.5 ± 116.0a | 0.89 ± 0.042a | 43.4 ± 6.5b | 0.099 ± 0.015b | 0.67 ± 0.039a |
|  | 600 | 365.0 ± 92.1a | 0.84 ± 0.047a | 41.2 ± 5.9b | 0.094 ± 0.013b | 0.63 ± 0.012b |

Data represent mean ± SEM (*n* = 4). Means followed by the same letters at column do not differ by the Scott-Knott clustering test (*p* > 0.05).
