# Supplementary material for: Growth, Physiology and Nutrient Use Efficiency in Eugenia dysenterica DC under Varying Rates of Nitrogen and Phosphorus
Source: Plants (Basel). 2020 Jun 8;9(6):722. doi: 10.3390/plants9060722 (PMC7355562; doi:10.3390/plants9060722)
Supplement: Supplementary file 1 [file plants-09-00722-s001.zip › Supplementary Material S3.docx]

**Table S3**. Height (H, cm), stem diameter (SD, mm), leaf number (LN), root length (RL, cm), total dry mass (TDM, g) and the ratio between shoot dry matter and root dry matter (SDM/RDM) in *Eugenia dysenterica* DC seedlings grown at varying rates of nitrogen (N, mg dm^−3^) and phosphorus (P, mg dm^−3^) for 278 d.

|  | | **H** | **SD** | **LN** | **RL** | **TDM** | **SDM/RDM** |
| --- | --- | --- | --- | --- | --- | --- | --- |
| N | 0 | 8.67 ± 0.50a | 2.26 ± 0.05a | 5.50 ± 0.28a | 71.75 ± 7.15a | 13.20 ± 0.74a | 0.14 ± 0.01a |
|  | 50 | 9.33 ± 0.56a | 2.44 ± 0.25a | 6.63 ± 1.04a | 73.00 ± 6.11a | 12.61 ± 2.26a | 0.23 ± 0.04a |
|  | 100 | 9. 24 ± 1.40a | 2.37 ± 0.18a | 6.50 ± 0.54a | 68.50 ± 4.17a | 12.60 ± 1.33a | 0.18 ± 0.04a |
|  | 200 | 8.16 ± 1.07b | 2.29 ± 0.17a | 5.13 ± 0.71b | 63.50 ± 9.20b | 11.78 ± 3.26a | 0.15 ± 0.03a |
|  | 400 | 7.86 ± 1.31b | 1.93 ± 0.27b | 6.13 ± 1.24a | 55.31 ± 6.75b | 6.02 ± 1.39a | 0.87 ± 0.63a |
|  | 0 | 6.88 ± 0.39b | 1.69 ± 0.20b | 2.75 ± 0.59b | 53.31 ± 5.52a | 8.41 ± 1.08a | 0.08 ± 0.01a |
|  | 100 | 8.68 ± 1.60a | 1.79 ± 0.22b | 5.00 ± 1.45a | 55.40 ± 4.22a | 8.72 ± 1.08a | 0.17 ± 0.03a |
| P | 200 | 10.1 ± 1.41a | 2.43 ± 0.27a | 5.00 ± 0.40a | 61.25 ± 2.48a | 12.4 ± 0.87a | 0.13 ± 0.03a |
|  | 400 | 10.9 ± 2.53a | 2.31 ± 0.42a | 4.88 ± 0.96a | 46.75 ± 2.59a | 12.03 ± 1.67a | 0.17 ± 0.05a |
|  | 600 | 9.36 ± 0.98a | 2.35 ± 0.23a | 5.50 ± 0.20a | 53.56 ± 5.22a | 11.29 ± 1.50a | 0.16 ± 0.02a |

Data represent mean ± SEM (*n* = 4). Means followed by the same letters at column do not differ by the Scott-Knott clustering test (*p* > 0.05).
